# Supplementary material for: Tead1a Initiates Transcriptional Priming Through the TEAD1a/YAP‐Notch1‐Spi1/Cebpα Axis to Promote Neutrophil Fate
Source: Adv Sci (Weinh). 2025 Aug 28;12(41):e05441. doi: 10.1002/advs.202505441 (PMC12591127; doi:10.1002/advs.202505441)
Supplement: Supplementary file 4 — Supplementary Dataset [file ADVS-12-e05441-s004.zip › 3-7.zip!/3-7/02-7.cluster/cluster18/Enrich/ego_CC.pdf]

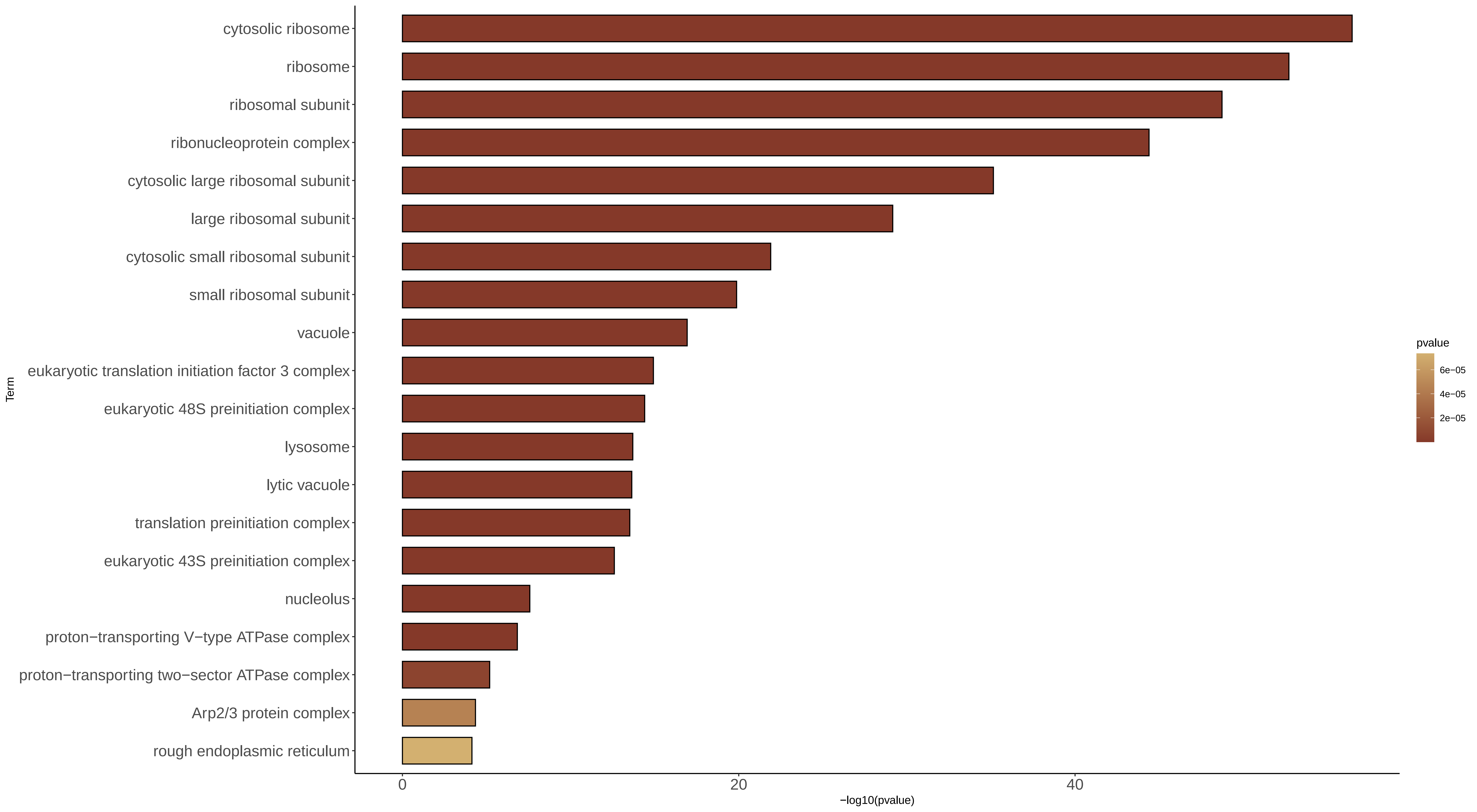
cytosolic ribosome
ribosome
ribosomal subunit
ribonucleoprotein complex
cytosolic large ribosomal subunit
large ribosomal subunit
cytosolic small ribosomal subunit
small ribosomal subunit
vacuole
eukaryotic translation initiation factor 3 complex
eukaryotic 48S preinitiation complex
lysosome
lytic vacuole
translation preinitiation complex
eukaryotic 43S preinitiation complex
nucleolus
proton−transporting V−type ATPase complex
proton−transporting two−sector ATPase complex
Arp2/3 protein complex
rough endoplasmic reticulum
Term
0
20
40
−log10(pvalue)
pvalue
6e−05
4e−05
2e−05
